# 

**Published:** 1819-03

**Authors:** 


### MONTHLY CATALOGUE OF MEDICAL BOOKS.

A Lecture on Dropsy; by George Gregory, M.D. 8vo. 2s.

An Appendix to the Pamphlet on the Early Symptoms of Water in the Brain; containing Cases successfully treated; with practical Illustrations of the Doctrines therein inculcated, and some Observations on the Functions of the Intestines, as connected with a Morbid Action of the Digestive Organs. By G. D. Yeats, M D. 8vo. 3s.

Practical Observations on the Treatment, Pathology, and Prevention, of Typhous Fever; by Edward Percival, M.B. M.R.I.A. &c. &c. 8vo. boards, 7s.

Remarks on Scepticism, especially as it is connected with the Subjects of Organization and Life; being an Answer to the Views of M. Bichât, Sir T. C. Morgan, and Mr. Lawrence, upon those Points. 8vo. 5s. 6d.

A Treatise on Midwifery; developing new Principles, which tend materially to lessen the Sufferings of the Patient, and shorten the Duration of Labour. By John Power, Accoucheur, &c. 8vo. 8s.

Facts and Observations relative to the Fever commonly called Puerperal; by John Armstrong, M.D. Second Edition, considerably enlarged. 8vo. 8s.

A Series of Engravings, representing the Bones of the Human Skeleton, with the Skeletons of some of the lower Animals; by Edward Mitchell, Engraver, Edinburgh. The explanatory References, by John Barclay, M.D.

An Essay on Fracture of the Patella, or Knee-pan; containing a new and efficacious Method of treating that Accident, by which the Deformity and Lameness that arise from the old and common Mode of Treatment are avoided; by John Sheldon, F.R.S. A new Edition, with additional Notes; by an Hospital Surgeon. 8vo. 2s. 6d.

The Elements of Natural Philosophy, illustrated throughout by Experiments which may be performed without regular Apparatus; by James Mitchell, M.A. 12mo. 8s.

